# Advertising in Medical Journals: Should Current Practices Change?

**DOI:** 10.1371/journal.pmed.0030130

**Published:** 2006-05-02

**Authors:** Adriane Fugh-Berman, Karen Alladin, Jarva Chow

## Abstract

Fugh-Berman and colleagues surveyed medical journals' policies and practices on advertising. Pharmaceutical products, they say, dominate journals' advertising pages, creating conflicts of interests.

Peer-reviewed medical journals are generally considered to be a source of unbiased and reliable information about drugs. But at the same time, most medical journals contain advertisements, almost all of which are for drugs, and which are, by their very nature, biased toward promoting sales of that drug. In this article, we address the purpose and effects of journal advertising, the possible reasons for pharmaceutical product domination of advertising pages, and the current policies and practices of representative journals. We also make recommendations for change.

## Advertising Drugs to Doctors: Scale and Impact

Advertisements are intended to increase or maintain market share for targeted products. Pharmaceutical companies value print advertisements because they increase sales effectively [ 1]. In a survey of 125 pharmaceutical marketers, journal advertising tied with detailing (pharmaceutical sales representative visits to doctors) as the most effective promotional and educational vehicle for “announcing a new product/new indication” [ 2]. Robert Osborn, President of the Dowden Health Media Journal Group (Montvale, New Jersey, United States of America), has been quoted as stating, “Advertising in medical publications is highly regarded by pharmaceutical marketers because it has the ability to generate rapid awareness based on its cost-efficient reach/frequency” [ 3].

In 2003, drug companies spent US$448 million on advertising in medical journals [ 4]. Scott Neslin, a marketing professor who conducted the Return on Investment Analysis of Pharmaceutical Promotion study, found that the overall return on investment (calculated as the average increase in revenues per incremental dollar spent in any given month) on journal advertisements was US$5.00. The range was US$2.22–US$6.86, with larger and older brands at the higher end (an executive summary of the study is available at http://www.rxpromoroi.org/rapp/index.html). Another industry study of 45 advertising campaigns found that journal advertising returned US$3.05 for advertising of unique products and US$2.46 for advertising of competitive established products (drugs that remain profitable despite competition—for example, sildenafil [Viagra], which remains profitable despite now sharing the erectile dysfunction market with tadalafil [Cialis] and vardenafil [Levitra]) [ 5].

Advertising increases prescriptions for targeted drugs in a dose-related manner.

Long-term returns may be even higher. While an advertisement in consumer magazines reaches potential purchasers individually, a pharmaceutical advertisement viewed by one physician could result in dozens or hundreds of drug purchases, especially if the drug is used over the long term. Although physicians believe that they prescribe based on impartial evidence, advertising has been shown to increase prescriptions for targeted drugs in a dose-related manner even when other promotional efforts are held constant [ 6].

Although consumer goods are primarily sold by advertising, pharmaceutical advertisements directed at physicians are an adjunctive influence rather than a primary influence—in other words, journal advertisements are rarely a physician's first exposure to a product, but are reminders designed to reinforce other promotional efforts. Besides detailing, other efforts include direct mail, electronic communications (sometimes called “E-detailing”), and promotional events. These events include overtly commercial “dinner talks,” satellite presentations (company-sponsored expert talks piggybacked onto medical society meetings), and continuing medical education conferences, usually handled through medical education companies.

Nevertheless, the pharmaceutical industry puts a high value on advertising its products in print journals. Milton Liebman, president of the Association of Medical Publishers (Westfield, New Jersey, United States of America), writes, “Print promotion usually provides higher message penetration than detailing because print (most notably journals) obtains higher reach into the target audience with great consistency of message delivery” [ 7]. Marshal Paul, chairman of PERQ/HCI Research (Princeton, New Jersey, United States of America), which conducts media research for the prescription drug industry, is quoted as stating, “Advertising magnifies the detailing effort at a fraction of detailing expense. In effect, detailing provides the power in the marketing effort and advertising provides the efficiencies” [ 5].

In an article called “A Medical Publisher Reminds Us: Don't Forget the Gatekeepers,” which discusses how advertising drugs to doctors can increase drug sales, Thomas Pizor writes that “the physician can thwart, with a single comment or stroke of the pen, all of the investment of a DTC [direct to consumer] campaign.” He suggests: “Let professional messages complement consumer messages. The copy platform and graphics should work together for all those times the patient will not remember the brand name but might remember the art or headline” [ 8]. The author notes that prescribers “stand between the consumer and your drug,” and so “it seems intuitive that DTC promotion of Rx-only [prescription-only] drugs will work only when the prescriber is treated as a central figure in the marketing plan.” It is clear, then, that the pharmaceutical industry views drug advertisements in journals as “third party advertisements,” directed not at the end users of the targeted product (i.e., patients), but at the physician as gatekeeper between drug companies and patients.

## Advertising Policies of Medical Journals: A Survey

It is clear why drug companies advertise in medical journals, but why do they dominate display advertising pages? In other words, why do journals appear to publish advertisements for drugs much more frequently than for cars, computers, vacations, or other products of potential interest to doctors?

We set out to explore this apparent monopoly by pharmaceutical companies of display (not classified) advertising pages. We began by looking at whether medical journals do in fact state a preference for publishing advertisements for drugs and medical devices over other kinds of products. First, we examined the advertising policies of nine multispecialty medical journals: the *New England Journal of Medicine (NEJM)*, the *Journal of the American Medical Association (JAMA), Annals of Internal Medicine (Annals), PLoS Medicine, American Family Physician*, the *Lancet*, the *BMJ*, the *Canadian Medical Association Journal*, and the *Medical Journal of Australia*. Multispecialty journals were chosen because they receive the most advertising revenue. According to PERQ/HCI, in 2002, US$220,982,000 was spent on advertising in multispecialty journals, almost ten times the amount spent on the runner-up, internal medicine journals (US$23,999,000) [ 9]. Advertising policies were obtained from the Web sites of journals or publishers, or by request.

Four of the five North American journals with advertising policies posted on Web sites accepted only advertisements relevant to medicine; *PLoS Medicine* (an international journal published in the United States) accepts no advertisements for drugs or medical devices. Of the journals surveyed, only the *Canadian Medical Association Journal* specifically mentioned consumer goods, stating, “Products or services eligible for editorial display advertising should be germane to medical practice, medical education, professional development or health care delivery. Consumer products and nonfinancial services that are offered by responsible advertisers and that are of interest to physicians may be eligible.” The *Lancet*, *BMJ*, and the *Medical Journal of Australia* did not address the relevance of product advertising in guidelines available on their Web sites (see sidebar).

*JAMA* states that “products or services eligible for advertising shall be germane to, effective in, and useful in (a) the practice of medicine, (b) medical education, and/or (c) health care delivery and shall be commercially available.” The *NEJM* states that “All advertising must be clearly germane to the practice of medicine.” *Annals* states that “advertising will be accepted for products or services directly relevant to the practice of medicine” (examples include prescription and nonprescription drugs, medical equipment, books, journals, and medical services). *American Family Physician* requires that products or services “be germane to and effective in the practice of medicine” and notes that advertisements for non-health-related commodities “will be accepted if they are determined to be in harmony with the stated purpose of the publication,” which is stated elsewhere as “to serve the medical profession and provide continuing medical education.”

Although no policy restricted advertising only to pharmaceuticals, in practice, drug ads dominate. We surveyed display advertisements from all January and June issues of *JAMA* from the last ten years in bound volumes at the Dahlgren Library, Georgetown University (Washington, D. C., United States of America). More than 95% of advertisements were for pharmaceutical products (see Table 1). The advertisements that were not for drugs were for health-related products, including medical books, medical Web sites, and computer products. Only military recruitment advertisements were unrelated to health care.

**Table 1:**
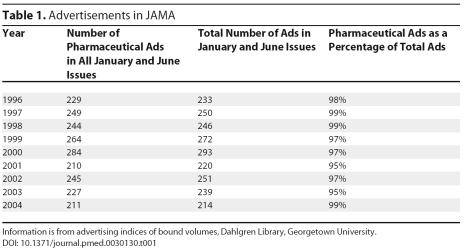
Advertisements in JAMA

Flipping through any peer-reviewed medical journal that accepts advertisements will show that the vast majority of advertisements are for prescription drugs; in the case of surgical journals, medical device ads are also prominent. A notable anomaly is the 1997 advertising menu of *JAMA*, which during that single year ran ads for Land Rover vehicles, Steinway pianos, and Wellcraft boats as well as for pork (“the other white meat”) and Quaker oatmeal. Intrigued by this apparent departure from usual advertising practice, we attempted to document what had inspired the expansion into consumer products and why it was abandoned; we interviewed *JAMA* editors and scanned public documents, but could not find answers to our questions.

## Is It Only Drug Companies That Can Afford to Place Advertisements in Journals?

Do display advertisements in medical journals feature mostly drugs because only pharmaceutical companies can afford such advertisements? Curious as to whether journal advertisements were beyond the means of consumer goods manufacturers, we compared advertising rates among journals affiliated with major medical societies against rates among several consumer magazines. We obtained rates and policies from publication Web sites or via E-mailed requests to advertising directors.

Surprisingly, general medical journal advertisements are far less expensive than magazine advertisements ( Table 2; data sources for Table 2 are shown in Box 1). Adjusting for circulation does not eliminate disparities. For example, although *Vogue'*s circulation of 1,150,000 is almost four times larger than *JAMA'*s circulation of 300,000, a *Vogue* advertisement costs almost seven times as much as a *JAMA* advertisement.

**Table 2:**
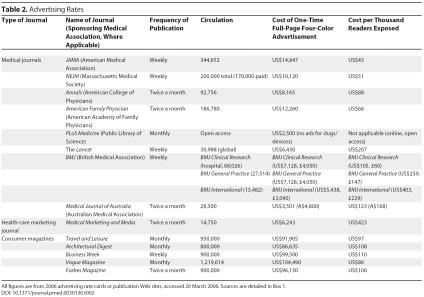
Advertising Rates

In 2004, *JAMA* and *NEJM*, the two largest and most influential US journals, had the highest revenues from advertising [ 10] and the cheapest advertising rates: a *JAMA* advertisement, only US$43 per thousand readers; an *NEJM* advertisement, only US$51 per thousand readers. In contrast, advertisements in consumer magazines range from US$86–US$110 per thousand readers ( Table 2).

Some medical journals trumpet their bargain rates. Journals solicit pharmaceutical advertising by placing their own advertisements in *Medical Marketing and Media (MMM)*, a publication aimed primarily at pharmaceutical and other health-care manufacturers. One *JAMA* advertisement in *MMM* states, “A priceless audience. At a price you can afford” (as seen in the June 2002 issue of *MMM*). An *Annals* advertisement states, “With an audience of more than 90,000 internists (93% of whom are actively practicing physicians), *Annals* has always been a smart buy” (as seen in the October 2000 issue of *MMM*).

Other advertisements placed by medical journals in *MMM* emphasize building relationships between doctors and drug company products. “Wisdom is for sharing,” is the headline of an advertisement from the *NEJM*, with a peculiar graphic of two people lying down, head to head, with what appears to be an egg between them. The advertisement goes on to say “Place your ad in the *New England Journal of Medicine* and make our relationship with the medical community yours” (as seen in the November 2003 issue of *MMM*). Another advertisement, with a graphic of autumn leaves, is headlined “You'll be *amazed* at what a *week* in *New England* can do,” and states, “Once a week, your advertising message is delivered to physicians who make decisions and influence the decisions of others.” And under the *NEJM* logo, the advertisement states, “The place to be for important news, important readers, important prescribers” (as seen in the October 2000 issue of *MMM*). An advertisement for the *Archives* journals states “Connect in an environment of trust and reap the benefits of relationship marketing” (as seen in the January 2003 issue of *MMM*).

In contrast, a four-page advertisement for the Endocrine Society, which publishes the *Journal of Endocrinology and Metabolism*, is all business. “The highly cited articles published in JCE&M discuss the application of your company's therapeutic products in the treatment of various endocrine disorders.” Other services are also touted: “we use our know-how to help you reach the industry's thought leaders and maximize your return via our medical journals, annual meetings, continuing education and corporate development programs. In addition to this, patient education programs provided by The Hormone Foundation, our public education and outreach affiliate, provide more opportunities for increased public exposure” (as seen in the May 2002 issue of *MMM*).

Advertising in *MMM* is no bargain. At US$423 per thousand exposed, the rate is higher than any other journal or magazine surveyed [ 11].

## Targeting Specific Groups of Physicians

Medical journals make prescribers available to pharmaceutical companies and offer a variety of ways of targeting particular prescribers. Financial arrangements between journals and pharmaceutical companies result in different ads being sent to different physicians—so a New York cardiologist and an Omaha pediatrician who subscribe to the same journal may receive distinct medleys of advertisements. This is made possible by “insert” advertisements, which are prepared by advertisers and attached to the journal after production. Insert advertisements, which are more expensive than the “film” advertisements printed with the journal content, enable advertisers to target “mini-demographics,” including those grouped by specialty, region, or most importantly, by prescribing proclivities.

Physicians who write the most prescriptions receive the most advertisements. The *NEJM* states “You can run your Cancer or HIV/AIDS related product to a special list of high-prescribing physicians, including key oncology and infectious disease doctors” [ 12]. *JAMA* also offers advertisers the chance to target “high-prescribing internal medicine physicians and other key specialists who prescribe AIDS-related [or ‘cancer-related’] products” through a special, addense version of *JAMA* that contains an average of “64 ad pages and 102 editorial pages” [ 13]. *Annals* states, “There are more internists in the United States (194,588 as of 2003) than family practitioners (97,051) and general practitioners (13,170) combined, and last year internists wrote nearly half a billion prescriptions. This group constitutes the ideal market for advertisers like you wishing to reach high-prescribing clinicians treating adult patients.” *Annals* also provides a list of the “top 20 diagnoses internists make” as well as the top 20 drug classes and top 20 brands prescribed by internists [ 14, 15]. And *American Family Physician* offers “prescription data available for therapeutic classes and products, profiled by physician specialty, prescription writing and revenue levels. Data can be provided in desired format to active and prospective advertisers” [ 16].

Research on journal advertisements is frustrating, because library journal issues usually contain only the film advertisements, not the insert ads placed in subscriber issues. Bound volumes have even fewer ads. In order to save shelf space, many libraries, including the US National Library of Medicine, have advertisements removed from journals when they are sent for binding.

## Advertising as “Education”

As a crucial part of their business model, many medical journals rely on revenue from prescription drug advertisements. And the argument has been made that advertisements provide doctors with valuable information about drugs and diseases [ 17]. But are drug advertisements really educational? Pharmaceutical advertisements may provide descriptive and prescribing information, but their primary purpose is to increase the sales of new products or maintain sales of established products. There are more than 10,000 drugs in the US market, but more than half of promotional expenditures are spent on the 50 best-selling drugs [ 18]. New drugs are emphasized.

New drugs are not always superior to old drugs, and best sellers are not always better than their competitors. If medical journals accepted only advertisements for drugs proven superior in comparative trials, the argument that drug advertisements are educational could be rationalized. No journal, however, requires demonstration of product superiority as a condition for advertising. Competing drugs regularly cohabit in the same journal (and when they do cohabit, they may be kept apart—the advertising rate card of the *Annals of Internal Medicine* states that “Competitive products are separated by no fewer than four pages for primary indication only, as reflected in the ad content.”

The “advertising-as-education” argument actually reached the US Supreme Court. In 1975, the American College of Physicians, publisher of *Annals*, argued that profits on pharmaceutical advertising should not be taxed because the advertisements were educational and thus related to the journal's tax-exempt status [ 19]. The US Claims court disagreed, noting that any educational function was incidental to revenue-raising function, and that “The evidence is clear that plaintiff did not use the advertising to provide its readers a comprehensive or systematic presentation of any aspect of the goods or services publicized. Those companies willing to pay for advertising space got it; others did not. Moreover, some of the advertising was for established drugs or devices and was repeated from one month to another, undermining the suggestion that the advertising was principally designed to alert readers of recent developments” [ 20].

Reversed by the Court of Appeals [ 21], the case went to the US Supreme Court, which reversed the decision of the appeals court [ 22]. Writing for the majority, Justice Thurgood Marshall wrote, “we are bound to conclude that the advertising in Annals does not contribute importantly to the Journal's educational purposes” [ 20].

Drug advertisements are not public service announcements. A review of 109 full-page pharmaceutical advertisements concluded that 44% would lead to improper prescribing if a physician relied only on the information presented in the advertisement [ 23]. A study by Cooper and Schriger of ten US journals found that 126 of 438 advertisements with medical claims contained no medical references. Of 312 advertisements with references, 55% cited journal articles and 19% cited “data on file” (proprietary information that companies are not obligated to provide to clinicians). Only 20% of references to data on file were available [ 24]. Two other studies found similar results: data availability for references in advertisements was 20% [ 25] and 26% [ 26]. Published studies cited by advertisements are more likely to be company-sponsored; in Cooper and Schriger's survey, 58% of original research cited was sponsored by or had an author affiliated with the product manufacturer, compared with 8% of references to original research within research articles [ 24].

## Can Medical Journals Wean Themselves off Drug Advertising?

Publishing is expensive, and medical journals obviously need a business model to be sustainable [ 27]. Advertisements are only a part of the financial relationship between pharmaceutical companies and most medical journals. Pharmaceutical companies are the primary purchaser of reprints, and may buy thousands of reprints if they are advantageous to the drugs that they manufacture. Additionally, drug companies “sponsor” subscriptions [ 28] provided without cost to targeted populations (referred to as “controlled” subscriptions). The recently deceased *BMJ USA* (a derivative publication of the weekly *BMJ*, containing selected articles, more advertisements than the weekly *BMJ*, and some US-specific content) was almost 100% controlled, with all but 25 of 95,000 subscriptions apparently sponsored by pharmaceutical companies. Most subscriptions were sent to high prescribers [ 29].

The *Lancet* tells potential purchasers of sponsored subscriptions that “three quarters of all respondents [recipients of sponsored subscriptions who responded to a Lancet-commissioned survey] were aware that their subscription to *The Lancet* was sponsored by a pharmaceutical company. Moreover, one half also recalled, on an **unaided** [bold text in the original quotation] basis, the name of the sponsoring company” [ 30]. “Arm your sales force with sponsored subscriptions to offer physicians on sales calls,” the *NEJM* suggests, promoting its “coverwrap, consisting of a customized cover and prime advertising pages, [which] delivers your product with your distinct message to thousands of physicians a week” ( http://nejmadsales.org/media/pdfs05/NEJMcoverwrap05.pdf). The *NEJM* also offers to “conduct complimentary research for you among sponsored subscribers to validate the success of your program” ( http://nejmadsales.org/incentives/sponsorship.html).

Journal advertisements generate profits not only for pharmaceutical companies and medical journals, but also for the physician organizations that publish the journals. In 1996, five of six physician organizations raised 10% or more of their total annual revenue from pharmaceutical company advertising in affiliated medical journals; the proportion of total revenues ranged from 2.1% to 31.3% [ 31]. In 2004, advertising in American Medical Association (AMA) publications constituted 15.1% of total AMA revenues [ 32]. The AMA received US$40.7 million from advertising in publications (presumably including *JAMA*, the *Archives* journals, and *American Medical News*), more than twice the US$17.5 million received from subscriptions [ 32].

By accepting only advertisements for drugs and medical devices, medical journals have accepted an exclusive and dependent relationship with corporations. Perhaps the distance between the prescribing physician and the purchase of the advertised product contributes to the perception that drug advertisements in journals are somehow more professional than advertisements for goods hawked directly to the consumer. However, all advertising campaigns are designed to create positive feelings about a brand, to saturate a targeted population's environment with a brand name, and to sell the product.

In consumer magazines, article content often caters to, or is ordered by, advertisers, and publishing articles that displease advertisers can financially jeopardize the publication. Medical journals are similarly prone to leash-yanking by advertisers.

A 1995 survey of North American journal editors found that 12% noted conflicts between advertisers' wishes and editorial decisions, and 21% reported that they had no control over advertisements [ 33]. In 1992, a study in *Annals* criticized the accuracy of advertisements in journals [ 23]. Several large pharmaceutical companies subsequently withdrew advertising in that journal [ 25], costing an estimated US$1 million to US$1.5 million in lost revenue [ 34]. Ensuing tension with the American College of Physicians contributed to the 1993 resignations of the coeditors, Robert and Suzanne Fletcher. In an interview, Robert Fletcher said, “The pharmaceutical industry showed us that the advertising dollar could be a two-edged sword, a carrot or a stick. If you ever wondered whether they play hardball, that was a pretty good demonstration that they do” [ 35].

At times, advertising departments appear to influence editorial decisions.

At times, advertising departments appear to influence editorial decisions. In 2001, the advertising department of *Annals* expressed concern about a planned editorial on high drug costs. These concerns were allayed when the editor invited a commentary from the Pharmaceutical Research and Manufacturers Association (Washington, D. C., United States of America), which commissioned a piece from a conservative thinktank, the American Enterprise Institute for Public Policy Research (Washington, D. C., United States of America) [ 35]. In 2004, an editor at *Dialysis and Transplantation* explained to an author that although the author's article had passed both editorial and peer review, the marketing department had rejected it [ 36]. The journal later reversed its decision, but the author refused to resubmit it, stating that “This episode has convinced us that the responsible action on our part is to continue our current efforts to have this manuscript published in a more academic journal” [ 37].

Several authors have suggested that dependence on drug advertising endangers the independence of medical journals [ 38] and have advocated other sources of revenue [ 20]. Although physicians would be expected to be a desirable audience for purveyors of cars, golf clubs, cruises, and luxury goods, advertisements for consumer goods in medical journals are conspicuous by their absence. Orentlicher and Hehir have argued compellingly that if advertisements for luxury goods were accepted, “journals would have a larger pool of companies to which they could sell advertising space, and they would reduce the conflict of interest that arises from the practice of only accepting health care advertisements. This suggests that health care companies are not the first place medical journals should look for advertising. Rather, they are the last place medical journals should look” [ 19].

Dependence on a single industry can be financially disastrous for a journal that displeases its corporate funders. Dependence on prescription drug advertising is also financially risky, as the journal risks closure if it can't attract sufficient revenue—indeed, *BMJ USA* has folded because “it has fallen victim to the widespread downturn in US pharmaceutical advertising and has become financially unsustainable for the BMJ Publishing Group” [ 39]. Accepting advertising for consumer goods removes the conflict of interest inherent in pharmaceutical advertising, but more importantly may free editors from the threat of lost revenue. It is disturbing that medical journals appear to have exclusive, largely undeclared arrangements with pharmaceutical companies. It could even be argued that it is poor business practice to forego more lucrative advertisements in order to provide cut-rate advertising to manufacturers of drugs and devices. If purveyors of consumer goods are willing to advertise in medical journals, replacing drug advertisements with advertisements for consumer goods could bolster both the bottom line and editorial freedom.

Another option is to eschew journal advertising altogether. Some journals refuse to advertise drugs or devices, depending instead on other sources of income. The non-profit journal *PLoS Medicine*, for example, uses a business model that includes support from foundations, from institutional memberships, and from asking research funders to pay a publication charge for accepted research papers ( http://www.plos.org/support/index.html).

## Conclusion

Clinicians rely on medical journals for scholarly articles and the latest information on drugs and devices. Advertisements in these journals are unreliable sources of information, since they “educate” physicians to prescribe the newest, most expensive drugs (which may not be any superior to existing, less expensive alternatives).

The scholarly nature of journals confers credibility on both articles and advertisements within their pages. By exclusively featuring advertisements for drugs and devices, medical journals implicitly endorse corporate promotion of the most profitable products. Advertisements and other financial arrangements with pharmaceutical companies compromise the objectivity of journals.

The primary obligation of industry is to make money for its stockholders. The primary obligation of journals should be to physicians and their patients, who depend on the accuracy of information within these publications. Medical journals should not accept advertisements from pharmaceutical companies, medical device companies, or other industries “relevant to medicine.”

**Box 1. Sources of Data for Table 2**

***Journal of the American Medical Association:*** http://pubs.ama-assn.org/misc/jamarates.pdf

***The New England Journal of Medicine:*** http://nejmadsales.org/media/pdfs06/06USRateCard.pdf; http://nejmadsales.org/incentives/advertisingopportunities/minidemo.html; http://nejmadsales.org/incentives/research.html#journal; http://nejmadsales.org/faq/index.html

***Medical Journal of Australia:*** http://www.mja.com.au/classifieds/Advtg_index.html

***BMJ:*** http://www.bmjpg.com/advertising

***Canadian Medical Association Journal:*** http://www.cmaj.ca/pdfs/cmaj.pdf

**The *Lancet:*** http://www.elsmediakits.com/ratecards/LANCET_display06.pdf

***American Family Physician:*** http://www.aafp.org/PreBuilt/afp_ratecard_2006.pdf

***Annals of Internal Medicine:*** http://www.acponline.org/journals/advert/annals_rates06.pdf

***PLoS:*** http://www.plos.org/support/index.html

***Medical Marketing and Media:*** http://www.mmm-online.com/content/fileadmin/files/mediainfo/onesheet.pdf

***Forbes:*** http://www.forbesmedia.com/forbes/forbrates.php

***Vogue:*** http://www.srds.com/listinglink/advertisinginfo/vogue

***Architectural Digest:*** http://www.condenastmediakit.com/ad/genrates.cfm

***Travel and Leisure:*** http://www.tlmediakit.com/nat_rates06.cfm

***Business Week:*** http://mediakit.businessweek.com/PDF

**Sources of Advertising Policies of Nine Multispecialty Medical Journals**

***New England Journal of Medicine:*** http://nejmadsales.org/media/pdfs06/06USRateCard.pdf; http://nejmadsales.org/incentives/sponsorship.html

***Medical Journal of Australia:*** http://www.mja.com.au/classifieds/Advtg_index.html

***Canadian Medical Association Journal:*** http://www.cmaj.ca/misc/advertising.shtml; http://www.cma.ca/index.cfm/ci_id/25274/la_id/1.htm#restrictions

***PLoS Medicine:*** http://medicine.plosjournals.org/perlserv/?request=get-document&doi=10.1371/journal.pmed.0010022; http://www.plos.org/support/index.html

***Annals of Internal Medicine:*** http://www.acponline.org/journals/advert/annals_rates06.pdf; http://www.acponline.org/journals/advert/pharm/start_acp.pdf

***American Medical Association:*** http://pubs.ama-assn.org/misc/adprinciples.pdf

***BMJ:*** http://www.bmjpg.com/advertising

**The *Lancet:*** http://www.elsmediakits.com/ratecards/LANCET_display06.pdf

***American Family Physician:*** http://www.aafp.org/PreBuilt/afp_ratecard_2006.pdf

All Web sites accessed between 2005 December 23 and 2006 January 3.

## Abbreviations

AMA: American Medical Association
*Annals*: *Annals of Internal Medicine*
*JAMA*: *Journal of the American Medical Association*
*MMM*: *Medical Marketing and Media*
*NEJM*: *New England Journal of Medicine*
