# Correction: Advertising in Medical Journals: Should Current Practices Change?

**DOI:** 10.1371/journal.pmed.0030303

**Published:** 2006-07-11

**Authors:** Adriane Fugh-Berman, Karen Alladin, Jarva Chow

In *PLoS Medicine*, volume 3, issue 6: DOI: 10.1371/journal.pmed.0030130

Table 2 should have included the following information about the CMAJ (Canadian Medical Association Journal): frequency of publication, twice a month; circulation, 70,000; cost of one-time full-page four-color advertisement, US$4,414 (CAD$4,900); cost per thousand readers exposed, US$63 (CAD$70).

